# Screening and characterization of thermostable enzyme-producing bacteria from selected hot springs of Ethiopia

**DOI:** 10.1128/spectrum.03710-23

**Published:** 2024-01-31

**Authors:** Meseret Guta, Genet Abebe, Ketema Bacha, Piet Cools

**Affiliations:** 1 Department of Biology, Jimma University, Jimma, Ethiopia; 2 Department of Diagnostic Sciences, Ghent University, Ghent, Belgium; State Key Laboratory of Microbial Resources, Institute of Microbiology, Chinese Academy of Sciences, , China

**Keywords:** enzyme screening, Ethiopia, hot springs, thermophiles, thermostable enzyme

## Abstract

Hot springs are potential sources of diverse arrays of microbes and their thermostable hydrolytic enzymes. Water and sediment samples were collected from three hot springs of Ethiopia and enriched on nutrient and thermus agar media to isolate pure cultures of potential microbes. A total of 252 bacterial isolates were screened and evaluated for the production of amylase, protease, cellulase, and lipase. About 95.23%, 84.12%, 76.58%, and 65.07% of the isolates displayed promising amylase, proteases, cellulase, and lipase activities, respectively. Based on the diameter of the clear zone formed, 45 isolates were further screened and identified to species level using matrix-assisted laser desorption/ionization time-of-flight-mass spectrometry analysis and 16S rRNA gene sequencing. Five of the 45 isolates showed significantly high (*P* < 0.05) clear zone ratios as compared to others. The identified isolates were categorized under five bacterial species, namely, *Bacillus licheniformis*, *Bacillus cereus*, *Paenibacillus thiaminolyticus*, *Paenibacillus dendritiformis*, and *Brevibacillus borstelensis*. The most dominant species (66.7%) was *B. licheniformis*. It could be concluded that hot springs of Ethiopia are potential sources of thermostable extracellular hydrolytic enzymes for various industrial applications. Further optimization of the growth conditions and evaluation for better productivity of the desired products is recommended before attempting for large-scale production of the hydrolytic enzymes.

**IMPORTANCE:**

Thermostable microbial enzymes play an important role in industries due to their stability under harsh environmental conditions, including extreme temperatures. Despite their huge application in different industries, however, the thermostable enzymes of thermophilic microorganism origin have not yet been fully explored in Ethiopia. Here, we explored thermophilic bacteria and their enzymes from selected hot spring water and sediment samples. Accordingly, thermophilic bacteria were isolated and screened for the production of extracellular hydrolytic enzymes. Promising numbers of isolates were found as producers of the enzymes. The potent enzyme producers were further identified using matrix-assisted laser desorption/ionization time-of-flight-mass spectrometry analysis and 16S rRNA gene sequencing. The findings revealed the presence of potential hydrolytic enzyme-producing thermophilic bacteria in hot springs of Ethiopia and necessitate further comprehensive study involving other extreme environments. Our findings also revealed the potential of Ethiopian hot springs in the production of thermostable enzymes of significant application in different industries, including food industries.

## INTRODUCTION

Thermal hot water springs (briefly, hot springs) are places where the water temperature is higher than that of the ambient temperature (1). Hot springs are formed by the emergence of geothermally heated groundwater from the Earth’s crust and found in regions of young volcanic activity (2). Hot springs can harbor diverse groups of microorganisms, and phenotypic differences of microorganisms in such environments are influenced by different physicochemical conditions, biogeography, and geological history of the area. It is very important to explore such environments for their microbial diversity from the conservation and biotechnological application points of view (3).

Extremophiles are microorganisms that survive in extreme environmental conditions. Hot springs are exceptional sites for microbial life and are characterized by their moderate-to-high temperature and regarded as one of the best sources of industrially important thermophiles (4). These thermophilic environments are places where the diversity of the three domains of microbes (i.e., archaea, bacteria, and eukaryotes) exists (5). Some of the hot spring microbial genera include *Sulfolobus*, *Acidiplasma*, *Caldivirga*, *Thermocladium*, and *Pyrobaculum*, all belonging to archaea. In fungi, *Rhizomucor*, *Chaetomium*, *Talaromyces*, and *Aspergillus* are the most commonly isolated genera, while bacteria are dominated by *Streptomyces*, *Pseudoxanthomonas*, *Geobacillus*, *Thermus*, *Thermoanaerobacter*, *Anoxybacillus*, and *Bacillus* (6).

Only thermophilic microorganisms are capable of growth and survival at higher temperatures. Thermophilies have shown remarkable potential for biotechnological applications because of their ability to produce unique thermostable enzymes and proteins, including amylases, cellulases, chitinases, pectinases, xylanases, proteases, lipases, and DNA polymerases. Thermostable bacterial and fungal enzymes have been increasingly applied to industrial sectors (7) and with high industrial and economic values (8). One of the classical examples of thermostable enzymes is Taq DNA polymerase, an enzyme purified from *Thermus aquaticus*, a bacterium isolated from hot springs (9), known for its major importance in PCRs.

Thermostable enzymes show unique features, such as they are usually not denatured at high temperatures but are rather more active at elevated temperatures (10). Moreover, they have been reported to be more stable against many solvents, detergents, and acidic and alkaline pH in comparison to their mesophilic counterparts (11).

In Ethiopia, despite the presence of many hot springs, there is very limited information on the diversity of thermophilic microorganisms and their thermostable enzymes. The selected four hot springs considered for this study are among the potential areas producing thermostable enzymes due to their unique features. However, these thermal springs are the least researched and are an underutilized natural resource in Ethiopia. Both the culture-dependent and the culture-independent microbial diversity studies on Ethiopian hot springs are not well addressed yet (12, 13). Therefore, the aim of the current study was to screen and characterize thermostable enzyme-producing thermophilic bacteria from selected hot springs of Ethiopia for their potential application in industries.

## MATERIALS AND METHODS

### Description of the study area

**Fig 1:**
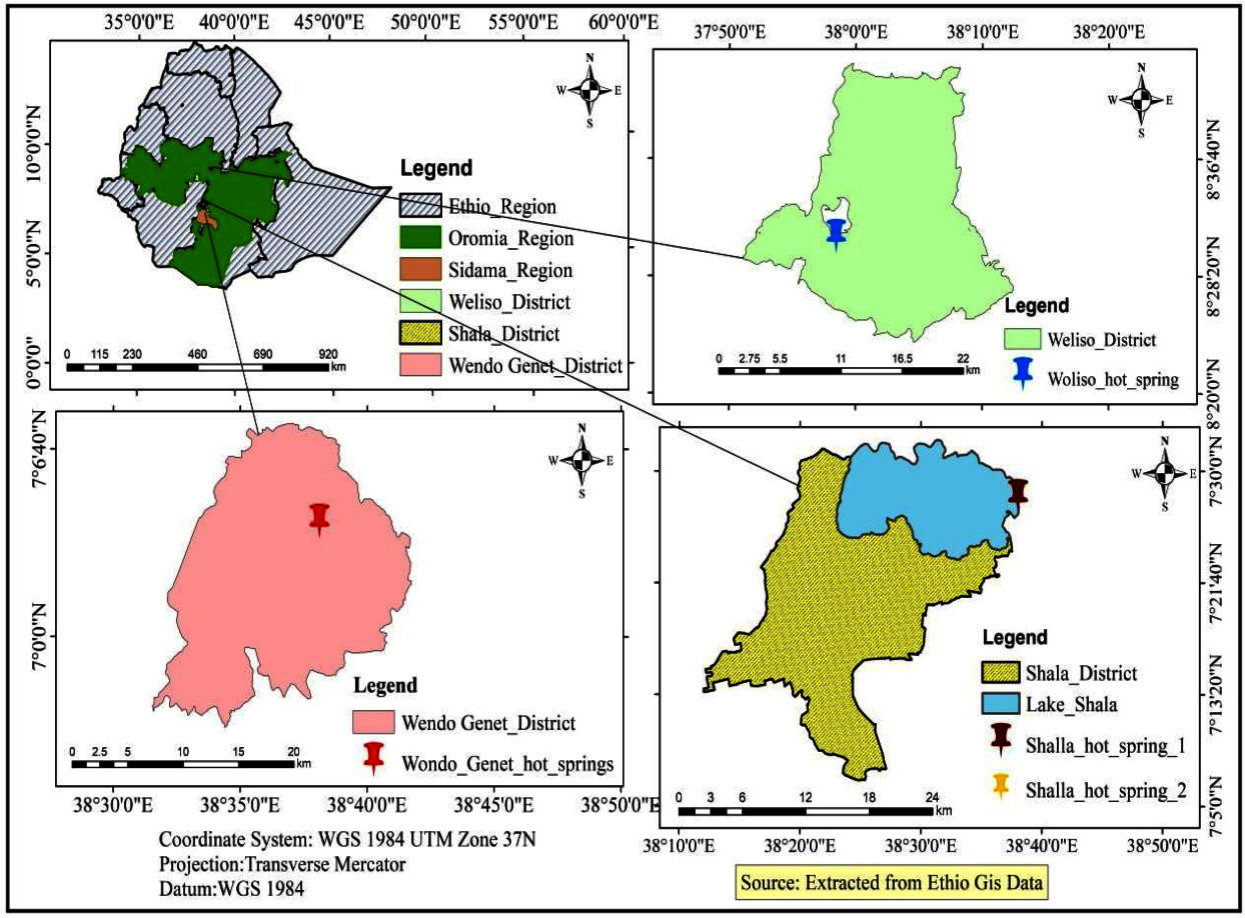
Map of the study sites.

The study was conducted at three hot spring locations in different geographical areas of Ethiopia: Woliso*,* Wondo Genet, and Shalla hot springs (Fig. 1). Woliso is the capital city of Southwest Shewa Zone, Oromia Regional State. The town is located at a distance of 114 km southwest of Addis Ababa. The geographical coordinates of the Woliso hot spring are 8°31′10″N and 37°58′27″E. Wondo Genet is a resort town in Ethiopia in the Sidama Regional State and is located at a distance of 236 km south of Addis Ababa. Wondo Genet is known for its hot springs, named after the town, which is located at 7°4′6590′N and 38°38′6780″E. The Shalla hot springs are some of the saline springs in Ethiopia, which are concentrated in the eastern and southwestern shores of Lake Shalla located some 286 km south of Addis Ababa. Two hot spring sites were selected for this study, one of which was located at 7°28′6880″N and 38°38′1070″E and the other at 7°28′6870″N and 38°38′1050″E.

### Sample collection

A total of 30 water and sediment samples (15 each) were collected from each of the four hot springs (Woliso*,* Wondo Genet, Shalla 1, and Shalla 2). For each of the four hot springs, three different sites were selected to collect the water and sediment samples. The water samples (500 mL) were aseptically collected using sterile thermal glass containers at three different depths (10, 20, and 30 cm below the surface) and away from the bank and immediately placed in a thermoflask to maintain the temperature of the water samples. Sediment samples (500 g) were also collected from the bottom of the hot springs using a sterile ladle and placed in sterile polythene bags. Then, the samples were aseptically transported to the Research and Postgraduate Laboratory (Jimma University, Jimma, Ethiopia) for further processing.

### Physicochemical properties of the hot springs

The temperature and pH of the hot springs were measured on-site by carefully immersing a digital portal thermometer and pH meter (pH-013, Hangzhou Langwei Technology Co., Ltd, China) in the hot spring, respectively. In addition, electrical conductivity (EC), dissolved oxygen (DO), and total dissolved solid (TDS) of hot spring water samples were also measured on-site and in the laboratory using a Palintest multiparameter photometer (Photometer 7500; Tyne and Wear, UK) following a method described earlier (14).

### Isolation of thermophilic bacteria

Thermophilic bacteria were isolated by transferring 25 mL of water or 25 g of sediment samples separately into 225 mL of sterile peptone water, and the suspension was homogenized at 100 rpm for 10 min in a homogenizer/shaker (Universal Shaker SM 30B, Edmund Buhler GmbH, Germany). After homogenization, the flasks were incubated for 24 h at the temperature of the respective hot springs (see Table 1). The incubation was also conducted at 45°C for all samples to increase the chances of isolation (15, 16). After incubation, a series of test tubes containing 9 mL of sterile peptone water were used for serially diluting the samples up to 10^−6^. Thereafter, a 0.1-mL aliquot of 10^−3^ dilutions of each sample was spread and plated on both nutrient agar (NA) and thermus medium (composition: 0.5% NaCl, 0.5% peptone, 0.4% beef extract, 0.2% yeast extract, and 2.0% agar) (17) and incubated at 45°C for 24–48 h. Single colonies with distinct morphology were selected from each plate and purified by successive streaking on respective fresh medium plates. Pure cultures were maintained at 4°C as NA slant and in nutrient broth with 20% glycerol stock at −20°C for further use.

**TABLE 1:** Physico-chemical characteristics of selected hot springs of Ethiopia

Parameter	Hot spring			
	Woliso	Wondo Genet	Shalla	
			Site 1	Site 2
Temperature (°C)	45 ± 0.00	71.9 ± 0.14	96.45 ± 0.35	88.15 ± 0.35
pH	7.75 ± 0.07	7.6 ± 0.00	8.1 ± 0.14	7.8 ± 0.14
EC (μS/cm)	1,225.5 ± 3.54	794.5 ± 3.54	9,247 ± 0.00	9,542.5 ± 3.54
TDS (mg/L)	791.5 ± 2.12	521 ± 1.41	5,094 ± 1.41	6,202.5 ± 3.54
DO (mg/L)	683 ± 2.83	676.5 ± 2.12	671.5 ± 2.12	657.5 ± 3.54
Salinity (ppm)	581.5 ± 2.12	341.5 ± 2.12	4,781 ± 1.41	5,380 ± 0.00

### Screening for extracellular hydrolytic enzymatic activities

Screening for the presence of four hydrolytic enzyme activities (amylase, lipase, protease, and cellulase) was conducted for all bacterial isolates. The isolates were grown on nutrient agar/thermus agar supplemented with respective enzyme-specific substrates for amylase, lipase, protease, and cellulase activities at 45°C for 24–48 h. Isolates exhibiting relatively larger clear zones around the colonies were selected as potent enzyme producers (18).

#### Amylase activity

Amylase activity was determined by growing isolates on a starch agar medium at 45°C for 24 h (19). After incubation, the plates were flooded with 1% Lugol’s iodine solution, and a clear zone formed around a colony indicated starch hydrolysis and hence a positive result for amylase production (19).

#### Cellulase activity

Nutrient agar supplemented with 1% carboxymethyl cellulose was used for the detection of cellulase-producing isolates. After incubation at 45°C for 24 h , the plates were flooded with an aqueous solution of Congo red (1% wt/vol) for 15 min. The Congo red solution was then discarded, followed by washing the plate with 1 M NaCl to detect the presence of clear halos around bacterial colonies, indicating cellulose degradation (16).

#### Protease activity

For the protease activity test, skimmed milk agar plates were prepared and solidified for 30 min, holes (3 mm diameter) were aseptically punched, and 10 µL of the enzyme solution, which was prepared by centrifugation at 10,000 rpm (4°C) for 20 min of an overnight activated culture, was injected into the hole. After 10 min, the plate was placed in an incubator at 45°C for 24 h. The transparent clear zone around the well was considered an indication of protease activity (20).

#### Lipase activity

Lipase activity was detected on nutrient agar medium supplemented with 1% Tween 80. The formation of an opaque halo around the colonies was considered positive lipolytic activity (16).

### Determination of clear zone ratio

For the determination of the clear zone ratio, 10 µL of an overnight activated culture (with cell density equivalent to 10^8^ cfu/mL) of each isolate was placed at the center of each enzyme-screening medium and incubated at 45°C for 24 to 48 h. Then, the diameters of the zone of clearance and of the colony were measured and used to calculate the clear zone ratio according to the formula of Latorre and colleagues (21).


$$Clear\;zone\;ratio\;(mm)=\frac{Diameter\;of\;zone\;of\;clearance}{Diameter\;of\;the\;bacterial\;colony}$$

Based on the results of the clear zone ratio, the isolates were categorized into excellent (clear zone ratio > 5.0), good (clear zone ratio > 2.0–5.0), or poor (clear zone ratio < 2.0).

### Conventional characterizations of the isolates

Morphological characterizations of the isolates with enzymatic activities were conducted after culturing the isolates on nutrient and thermus agar/broth at 45°C. Colony and cell morphologies of the isolates were assessed by direct observation of a distinct colony and microscopic observation of a single cell, respectively. Furthermore, the isolates were characterized for their Gram staining reaction (22), spore formation ability (23), and motility (24) following standard microbiological procedures. Citrate was used as the carbon source following a method described by Maina (25). The indole test was performed using Kovac’s reagent on bacterial growth on sulfide indole motility medium, and the formation of a red-colored ring was considered indole positive (26). Oxidase and catalase activities were performed as described earlier by Kovacs (27) and Maugeri et al. (28), respectively.

The optimum growth temperature and pH range were determined by incubating the isolates aerobically on nutrient agar at different temperatures ranging from 45°C to 70°C with 5°C intervals and pH ranging from 3 to 11 with one pH unit intervals. To determine the ability of the isolates to grow at different NaCl concentrations, isolates were also cultured on nutrient agar supplemented with 3%, 5%, 7%, 10%, 12%, and 15% (wt/vol) NaCl and incubated at 45°C, and growth was assessed after 24–48 h (29).

### Strain shipment and re-culturing

A total of 45 bacterial isolates, tentatively identified to the genus *Bacillus,* were transported to Ghent University, Belgium, for further identification by means of matrix-assisted laser desorption/ionization time-of-flight-mass spectrometry (MALDI-TOF-MS) and/or 16S rRNA sequencing as described earlier (30). Pure isolates of bacteria were transferred to sterile nutrient broth in 5-mL vials and incubated overnight; these were transferred to 2-mL Eppendorf tubes and transported to Belgium. Upon arrival, the isolates were transferred to fresh nutrient broth for re-culturing. After incubation, the bacterial isolates were grown on nutrient agar to check the viability of the isolates. Once checked for purity, the isolates were further identified by MALDI-TOF-MS and/or 16S rRNA sequencing.

### MALDI-TOF-MS identification

Isolates were identified using MALDI-TOF MS (Bruker Daltonik Microflex LT/SH) by both direct bacterial transfer and using the ethanol/formic acid extraction method as described previously (30). Isolates with no or poor identification (i.e., MALDI-TOF-MS score of <1.7) were further identified by means of 16S rRNA sequencing.

### Identification with 16S rRNA gene sequencing

DNA was extracted from the bacterial isolates using a crude alkaline lysis method as previously described (31). Then, the DNA extracts were used as a template for amplification of the 16S rRNA gene using the forward primer abNOT (5′-AGTTTGATCCTGGCTCAG-3′) and a reverse primer omegaMB (5′-TACCTTGTTACGACTTCGTCCCA-3′). PCRs were carried out in a total volume of 50 µL containing the DNA template, PCR-grade water, and final concentrations of FastStart PCR master Roche (1×), forward primer (0.2 µM), and reverse primer (0.2 µM). The PCR amplification conditions consisted of initial denaturation at 95°C for 5 min, followed by 35 cycles of denaturation at 95°C for 20 s, annealing at 50°C for 1 min, extension at 72°C for 1 min, and a final polymerization step of 72°C for 5 min. The amplified 16S rRNA gene products were loaded onto 1% (wt/vol) agarose gel, and electrophoresis was run at 180 V for 30 min before it was visualized under UV light having added ethidium bromide.

The PCR products were sent to GATC Eurofins for sequencing using abNOT and omegaMB as sequencing primers. The 16S rRNA gene sequences were edited with BioEdit 7.2 software and aligned to closely related bacterial species available at the GenBank database using the BLASTn program. Bacterial isolates were identified based on the highest percentage of sequence similarity to sequences already available in the GenBank.

### Data analysis

The results of enzymatic activity and physicochemical properties of the hot springs were expressed as the mean ± SD of the three different independent replicates. Analysis of variance followed by Duncan’s post hoc multiple comparison tests was done to compare the enzymatic activities of the isolates using SPSS software (version 26). Values with *P* ≤ 0.05 were considered statistically significant.

## RESULTS

### Physicochemical properties of the hot springs

The physical and chemical properties of water samples from the hot springs are shown in the table given below (Table 1). The results indicated that the Woliso hot spring had the lowest temperature of 45 ± 0.00°C, while Shalla hot springs had the highest temperature (96.45 ± 0.35°C). The Wondo Genet hot spring had the lowest pH (7.6 ± 0), while Shalla 1 had the highest pH of 8.1 ± 0.14. Shalla has a distinct feature of high salinity (4,781 ± 1.41–5,380 ppm) as compared to both Woliso (5,81.5 ± 2.12) and Wondo Genet (341.5 ± 2.12) (Table 1).

### Isolation, screening, and conventional characterization of hydrolytic enzyme-producing bacteria

A total of 252 bacterial isolates were isolated from water and sediment samples of the four hot springs based on morphological differences of their colonies. From the four hot springs, the highest number of isolates, i.e., 112 (44.4 %), was obtained from Woliso, 85 (33.7%) from Wondo Genet, and the rest 55 (21.8%) were isolated from the two Shalla hot spring sites. Bacterial isolates from sediment samples of all hot springs accounted for 53.6% (135/252) of the isolates, while water samples contributed to 46.4% (117/252) of the isolates.

Most of the 252 bacterial isolates (95.2%) showed amylase activity, while 212 isolates (84.1%), 122 isolates (76.6%), and 164 isolates (65.1%) produced proteases, celluloses, and lipases, respectively. All isolates produced at least one extracellular hydrolytic enzyme. Production of more than one enzyme was observed among 244 (96.8%) isolates. From the total isolates, 41.6% of the isolates exhibited activities for all the four hydrolytic enzymes, while 40.8%, 14.4%, and 3.2% were positive for activities of three enzymes, two enzymes, and one enzyme, respectively (data not shown).

Enzyme-producing isolates were different based on their morphological and biochemical characteristics. They were either brown, creamy white, whitish yellow, or yellowish white in color on NA. The colonies were either circular or irregular in shape with entire to undulate margins or erose to lobate edges. Most of the isolates had either smooth mucous, rough dry, or shiny mucous textures. They were either opaque or translucent. The biochemical test results showed that all 45 bacterial isolates were positive for catalase, while 37 were oxidase-positive, while two isolates were indole producers with only four isolates utilizing citrate as a carbon source. Furthermore, all the 45 isolates were Gram-positive, aerobic endospore producers, and motile, tentatively identified as *Bacillus* spp.

There were clear zone ratio differences among the isolates, reflecting the presence of higher/lower enzymatic activities. Based on the highest clear zone formation, 45 bacterial isolates were selected (Table 2). These isolates were further identified using MALDI-TOF MS and 16S rRNA sequencing. From the selected 45 bacterial isolates, five isolates showed a significantly different (*P* ≤ 0.05) clear zone ratio than the others (Table 2). Regarding amylase activity, isolates JUW103 and JUS2 revealed clear zone ratios of 4.92 ± 1.13 and 4.58 ± 0.73, respectively, and they were recognized as excellent amylase producers. In the case of protease, isolate JUS51 (4.86 ± 0.48) showed the highest clear zone ratio. The clear zone ratio of cellulase was the highest for isolate JUWG44 (4.35 ± 0.63), while the clear zone ratio of lipase was the highest for the isolate JUWG9 (4.52 ± 0.41).

**TABLE 2:** Biochemical characteristics and clear zone ratio (mm) (mean ± SD) of selected bacterial isolates from Ethiopian hot springs^*a*^

No.	Hot spring	Sample source	Species	Strain	Oxidase	Citrate	Indole	Amylase	Protease	Cellulase	Lipase
1	Woliso	Water	*Bacillus cereus*	JUW12	+	−	−	1.63 ± 0.2^j^	2.8 ± 0.3^f^	2.72 ± 0.15^f^	1.66 ± 0.15^gh^
2	Woliso	Water	*Paenibacillus thiaminolyticus*	JUW28	+	−	+	1.42 ± 0.16^j^	1.58 ± 0.08^gh^	3.33 ± 0.17^cdef^	3.51 ± 0.24^cd^
3	Woliso	Water	*Paenibacillus thiaminolyticus*	JUW29	+	−	−	3.61 ± 0.36^cdefgh^	1.57 ± 0.21^gh^	3.81 ± 0.37^abcd^	1.59 ± 0.14^h^
4	Woliso	Water	*Bacillus licheniformis*	JUW38	+	−	−	3.79 ± 0.19^cdefg^	1.66 ± 0.21^gh^	3.83 ± 0.44^abcd^	1.72 ± 0.1^gh^
5	Woliso	Water	*Paenibacillus thiaminolyticus*	JUW43	−	−	−	4.09 ± 0.44^bcde^	1.6 ± 0.23^gh^	2.96 ± 0.51^ef^	3.74 ± 1.22^bcd^
6	Woliso	Water	*Paenibacillus dendritiformis*	JUW86	+	−	+	3.76 ± 0.47^cdefg^	3.67 ± 0.52^cde^	1.4 ± 0.3^g^	1.5 ± 0.3^h^
7	Woliso	Water	*Paenibacillus thiaminolyticus*	JUW103	−	−	−	4.92 ± 1.13^a^	1.38 ± 0.14^h^	1.64 ± 0.2^g^	4.47 ± 0.97^ab^
8	Woliso	Sediment	*Bacillus licheniformis*	JUW54	+	−	−	4.3 ± 0.75^abc^	3.57 ± 0.6^cde^	1.51 ± 0.14^g^	1.63 ± 0.24^h^
9	Woliso	Sediment	*Bacillus licheniformis*	JUW62	+	+	−	1.41 ± 0.26^j^	1.5 ± 0^jh^	3.93 ± 0.7^abc^	4.13 ± 0.36^abc^
10	Woliso	Sediment	*Paenibacillus thiaminolyticus*	JUW64	+	−	−	1.44 ± 0.16^j^	3.4 ± 0.57^def^	1.53 ± 0.29^g^	3.64 ± 0.2^cd^
11	Wondo G.	Water	*Bacillus licheniformis*	JUWG3	+	+	−	1.68 ± 0.3^j^	1.44 ± 0.31^h^	1.43 ± 0.35^g^	3.83 ± 0.43^abcd^
12	Wondo G.	Water	*Bacillus licheniformis*	JUWG7	+	−	−	1.38 ± 0.13^j^	3.63 ± 0.63^cde^	1.73 ± 0.2^g^	1.52 ± 0.17^h^
13	Wondo G.	Water	*Bacillus licheniformis*	JUWG8	+	−	−	1.65 ± 0.08^j^	1.5 ± 0.07^gh^	1.62 ± 0.28^g^	3.27 ± 0.15^de^
14	Wondo G.	Water	*Paenibacillus thiaminolyticus*	JUWG9	+	−	−	1.65 ± 0.28^j^	1.61 ± 0.11^gh^	1.63 ± 0.23^g^	4.52 ± 0.41^a^
15	Wondo G.	Water	*Bacillus licheniformis*	JUWG21	+	−	−	3.98 ± 0.42^bcdef^	1.59 ± 0.35^gh^	1.46 ± 0.23^g^	3.44 ± 0.51^cd^
16	Wondo G.	Water	*Paenibacillus thiaminolyticus*	JUWG22	+	−	−	3.14 ± 0.25^ghi^	2.19 ± 0.07^g^	1.39 ± 0.18^g^	3.33 ± 0.48^cde^
17	Wondo G.	Water	*Bacillus licheniformis*	JUWG28	+	−	−	3.27 ± 0.29^fghi^	1.7 ± 0.47^gh^	3.74 ± 0.94^abcd^	1.37 ± 0.1^h^
28	Wondo G.	Water	*Bacillus licheniformis*	JUWG44	+	−	−	1.71 ± 0.24^j^	1.88 ± 0.4^gh^	4.35 ± 0.63^a^	1.32 ± 0.1^h^
19	Wondo G.	Water	*Bacillus licheniformis*	JUWG45	+	−	−	2.85 ± 0.17^i^	1.66 ± 0.15^gh^	1.4 ± 0.13^g^	3.47 ± 0.32^cd^
20	Wondo G.	Water	*Paenibacillus thiaminolyticus*	JUWG50	+	−	−	1.5 ± 0.07^j^	1.66 ± 0.15^gh^	3.56 ± 0.51^bcde^	2.45 ± 0.24^fg^
21	Wondo G.	Water	*Bacillus licheniformis*	JUWG51	−	−	−	1.47 ± 0.32^j^	1.96 ± 0.18^gh^	1.45 ± 0.21^g^	3.46 ± 0.36^cd^
22	Wondo G.	Water	*Bacillus licheniformis*	JUWG53	+	−	−	1.64 ± 0.19^j^	1.45 ± 0.21^h^	1.71 ± 0.37^g^	3.76 ± 1.08^bcd^
23	Wondo G.	Water	*Paenibacillus dendritiformis*	JUWG60	+	−	−	1.85 ± 0.33^j^	1.77 ± 0.07^gh^	3.67 ± 0.08^abcde^	1.5 ± 0.22^h^
24	Wondo G.	Water	*Bacillus licheniformis*	JUWG77	+	−	−	1.92 ± 0.2^j^	4.02 ± 0.42^bcd^	4.1 ± 0.53^ab^	1.44 ± 0.17^h^
25	Wondo G.	Water	*Bacillus licheniformis*	JUWG84	−	−	−	3.88 ± 0.55^bcdefg^	2.81 ± 0.35^f^	1.25 ± 0^g^	1.6 ± 0.31^h^
26	Shalla	Water	*Bacillus licheniformis*	JUS27	+	−	−	1.72 ± 0.1^j^	3.8 ± 0.42^cde^	1.68 ± 0.31^g^	1.75 ± 0.07^gh^
27	Shalla	Water	*Bacillus licheniformis*	JUS50	−	−	−	3.42 ± 0.36^efghi^	1.56 ± 0.12^gh^	3.47 ± 0.6^bcde^	1.66 ± 0.21^gh^
28	Shalla	Sediment	*Bacillus licheniformis*	JUS2	+	−	−	4.58 ± 0.73^ab^	1.89 ± 0.49^gh^	1.45 ± 0.21^g^	3.24 ± 0.45^de^
29	Shalla	Sediment	*Bacillus licheniformis*	JUS3	+	−	−	3.47 ± 0.19^defghi^	4.19 ± 0.46^bc^	3.51 ± 0.55^bcde^	1.36 ± 0.12^h^
30	Shalla	Sediment	*Bacillus licheniformis*	JUS4	−	−	−	1.73 ± 0.14^j^	4.65 ± 0.32^ab^	1.45 ± 0.21^g^	2.6 ± 0.24^ef^
31	Shalla	Sediment	*Bacillus licheniformis*	JUS5	−	−	−	3.62 ± 0.52^cdefg^	3.71 ± 0.14^cde^	3.33 ± 0.08^cdef^	1.6 ± 0.31^h^
32	Shalla	Sediment	*Bacillus licheniformis*	JUS9	+	−	−	1.6 ± 0.31^j^	1.59 ± 0.23^gh^	1.81 ± 0.32^g^	3.86 ± 0.25^abcd^
33	Shalla	Sediment	*Bacillus licheniformis*	JUS10	+	+	−	1.51 ± 0.11^j^	1.51 ± 0.38^gh^	3.32 ± 0.33^cdef^	1.6 ± 0.35^h^
34	Shalla	Sediment	*Bacillus licheniformis*	JUS14	+	−	−	3.91 ± 0.56^bcdefg^	4.11 ± 0.43^bc^	1.59 ± 0.14^g^	1.39 ± 0.28^h^
35	Shalla	Sediment	*Paenibacillus thiaminolyticus*	JUS28	+	−	−	1.72 ± 0.1^j^	3.8 ± 0.42^cde^	1.68 ± 0.31^g^	1.75 ± 0.07^gh^
36	Shalla	Sediment	*Bacillus licheniformis*	JUS33	+	−	−	1.81 ± 0.34^j^	1.59 ± 0.22^gh^	1.45 ± 0.21^g^	3.33 ± 0.33^cde^
37	Shalla	Sediment	*Bacillus licheniformis*	JUS38	+	−	−	2.96 ± 0.31^hi^	1.51 ± 0.14^jh^	1.24 ± 0.12^g^	1.38 ± 0.14^h^
38	Shalla	Sediment	*Paenibacillus thiaminolyticus*	JUS39	+	−	−	1.75 ± 0.23^j^	3.24 ± 0.55^ef^	3.38 ± 0.54^cdef^	1.44 ± 0.25^h^
39	Shalla	Sediment	*Brevibacillus borstelensis*	JUS42	+	−	−	1.6 ± 0.31^j^	1.48 ± 0.08^gh^	1.81 ± 0.22^g^	3.61 ± 0.35^cd^
40	Shalla	Sediment	*Bacillus licheniformis*	JUS49	+	+	−	4.22 ± 0.91^abcd^	4.07 ± 0.81^bcd^	1.44 ± 0.48^g^	1.96 ± 0.7^fgh^
41	Shalla	Sediment	*Bacillus licheniformis*	JUS51	−	−	−	3.79 ± 0.44^cdefg^	4.86 ± 0.48^a^	1.75 ± 0.4^g^	1.69 ± 0.39^gh^
42	Shalla	Sediment	*Paenibacillus thiaminolyticus*	JUS52	+	−	−	3.32 ± 0.33^efghi^	1.38 ± 0.14^h^	3.14 ± 0^def^	1.9 ± 0.16^fgh^
43	Shalla	Sediment	*Bacillus licheniformis*	JUS53	+	−	−	3.8 ± 0.42^cdefg^	1.33 ± 0.2^h^	3.2 ± 0.35^def^	3.68 ± 0.67^cd^
44	Shalla	Sediment	*Bacillus licheniformis*	JUS54	+	−	−	1.51 ± 0.24^j^	3.62 ± 0.52^cde^	3.72 ± 0.1^abcd^	1.7 ± 0.31^gh^
45	Shalla	Sediment	*Bacillus licheniformis*	JUS55	+	−	−	1.51 ± 0.14^j^	3.67 ± 0.63^cde^	3.98 ± 0.54^abc^	1.75 ± 0.29^gh^

^*a*^ Initials used to label isolates were based on initials of the hosting university, JU (Jimma University), followed by initials of the study sites, W (Woliso), WG (Wondo Genet), or S (Shalla). Mean values within the column followed by the same letter are not significantly different (*P* ≥ 0.05).

### Physiological characteristics of the selected isolates

Physiological parameters such as tolerance to temperature, pH, and NaCl were assessed to check for the optimal growth requirements of the 45 bacterial isolates. All isolates were able to grow within a temperature range of 45–55°C. Most (88.9%) of the isolates grew at 60°C, while more than a quarter (28.9%) grew at both 65°C and 70°C. Concerning acid tolerance, all isolates grew within the pH range of 6–8, while 28.9% and 17.8% of the isolates grew at pH 5 and pH 4, respectively. In addition, isolates grew best at 3% to 10% NaCl (wt/vol) concentrations, while 6.7% of the isolates could not grow at 12% NaCl. Even if some isolates grew under extreme conditions, it was observed that the temperature range between 50˚C and 55°C was the optimal growth temperature, while 7–8 was the optimal growth pH, and 3–10% was the optimal growth concentration of NaCl.

### Identification of the bacterial isolates using MALDI-TOF MS and 16S rRNA sequencing

The MALDI-TOF-MS-based identifications identified 43 of the isolates, among which 30 (69.8%) isolates were identified as *Bacillus licheniformis*, 11 (25.6%) as *Paenibacillus thiaminolyticus*, one (2.3%) as *Bacillus cereus,* and one (2.3%) as *Brevibacillus borstelensis*. Two of the bacterial isolates that could not be identified with MALDI-TOF-MS (score below 1.7), both with direct transfer and protein extraction method, were subjected to identification by 16S rRNA sequencing. The 16S sequence identified these isolates as *Paenibacillus dendritiformis* sharing 100% nucleotide sequence similarity with the sequences within the GenBank database (accession numbers MN055964 and MH027386). Among the isolates identified with both methods, the genus *Bacillus* dominated the isolates and accounted for about 71.1%, followed by *Paenibacillus* (26.7%). The least identified genus was *Brevibacillus* (2.2%) (Table 3). Among the members of the genus *Bacillus*, the most dominant species was *B. licheniformis,* which accounted for 30 (66.7%) of the isolates. Eleven isolates (24.4%)) were identified as *P. thiaminolyticus*. Furthermore, two of the isolates were related to *P. dendritiformis*, while one isolate each was identified to the species *B. borstelensis* and *B. cereus* (Table 3).

**TABLE 3:** Frequency distribution of dominant thermo-stable enzyme-producing bacterial species isolated from selected hot springs (*n* = 45) of Ethiopia

Sl no.	Bacterial species	Frequency of isolation (%)	Sample source	Identification method
1	*Bacillus licheniformis*	30 (66.7)	Water, sediment	MALDI-TOF-MS
2	*Paenibacillus thiaminolyticus*	11 (24.4)	Water, sediment	MALDI-TOF-MS
3	*Bacillus cereus*	1(2.2)	Water	MALDI-TOF-MS
4	*Paenibacillus dendritiformis ClaCZ203*	1(2.2)	Water	16S rRNA
*5*	*Paenibacillus dendritiformis BDU SRS2*	1(2.2)	Water	16S rRNA
6	*Brevibacillus borstelensis*	1(2.2)	Sediment	MALDI-TOF-MS

Of the total thermo-stable enzyme-producing bacterial isolates, *B. licheniformis* was the most frequently encountered isolate (66.7%), followed by *P. thiaminolyticus* (24.4%), while the others, including *B. borstelensis* and *B. cereus*, were rarely identified (2.2% each) in the study area (Table 3).

## DISCUSSION

The physical and chemical properties of water samples obtained from four hot springs were examined *in situ*. Accordingly, Shalla hot springs had a temperature of 96.45°C ± 0.35°C, while Woliso had a minimum temperature of 45°C ± 0°C. All the hot springs had from neutral to slightly alkaline pH (7.6 ± 0 to 8.1 ± 0.14). Similarly, Sahay et al. (15) reported that Manikaran hot springs located in the Indian Himalayas had a temperature ranging between 89°C and 95°C, while that of Yumthang hot springs had a temperature recorded between 43°C and 63°C and pH ranging between 7.8 and 8.2. In line with the current study, Alrumman and colleagues (32) also reported that the lowest and highest pH values recorded from the hot springs in Saudi Arabia were 7.2 and 8.2, respectively, while the temperature scale observed from the hot springs ranged from 60°C to 70°C with the minimum temperature of 55°C. The variation in temperature and pH values among the hot springs could be due to the differences in the physical conditions and chemical compositions of the hot springs. Such a difference could possibly account for the differences in the hot springs’ capacity to support the growth and survival of thermophilic bacteria (33).

The highest electric conductivity (9,542 ± 3.54 μS/cm) and TDS (6,202.5 ± 3.54 mg/L) were recorded from Shalla hot spring 2. In contrast to the current study, Alrumman et al. (32) reported lower electric conductivity (5,100 μS/cm) and TDS (3,147 mg/L) from a hot spring in Saudi Arabia. Similar to the current study, Panosyan et al. (34) also reported the highest EC value (6,722.9 μS/cm) from one of the hot springs located within the territory of Armenia. The high EC could be due to the presence of high mineral concentrations and organic materials, whereas the highest TDS may be due to the presence of inorganic salts. Elements such as calcium, magnesium, potassium, sodium, bicarbonate, chlorides, and sulfates as well as small amounts of dissolved organic materials increase the TDS of hot spring water (35). Furthermore, a high concentration of DO (683 ± 2.83) was recorded from Woliso hot spring, which had a lower temperature (45°C ± 0°C) compared to that of the other three hot springs in this study. This might be due to the fact that water temperature is inversely proportional to the concentration of DO. As the water temperature increases, the amount of DO decreases (36).

In the current study, 95.2%, 84.1%, 76.6%, and 65.1% of the bacterial isolates were amylase, protease, cellulase, and lipase producers, respectively. Similarly, Lele and Deshmukh (37) reported that all bacterial isolates from Indian hot springs were amylase producers, but percentages of protease, cellulase, and lipase producers were 60%, 20%, and 20%, respectively, which is lower than the percentage of producers recorded in this study. In line with our study, Mohammad and co-workers (16) and Aanniz et al. (38) also reported that the amylase-producing ability of bacteria isolated from Jordanian and Moroccan hot springs was very high. In their reports, it was indicated that 70% and 71.25% of the isolates were amylase producers, respectively. Furthermore, Yadav and co-workers (39) reported related results, in which the majority of the isolates (67%) were amylase producers, while 63% were cellulase producers, but the lipase-producing ability of the isolates was much lower (10%) than that in the current study. On the contrary, Walid Elkazzaz et al. (40) encountered a high number of bacterial isolates with cellulase activity. According to their report, the majority (95%) of the isolates were cellulase producers. In line with the current study, Benammar et al. (41) also reported a comparable percentage (64.50%) of lipase-producing isolates from the hot springs of Algeria. The reason for the differences in enzyme production might be the physical and chemical properties of hot spring waters. Environmental conditions and the chemical properties of hot springs also cause a significant difference in enzyme production (32). The increased production of amylase from bacteria makes the center of attention to different industries. The bulk production of amylases from microorganisms is economical, and this makes them candidates for use in different industries (42).

In the current study, all the 252 bacterial isolates showed at least one extracellular hydrolytic enzyme activity. Similar to this study, Benammar and co-workers (41) also indicated that among the 293 thermophilic bacteria, 99.32% of them produced at least one extracellular hydrolytic enzyme. Combined activity for more than one enzyme was also observed among the majority of bacterial isolates in which 41.6%, 40.8%, and 14.4% of the isolates exhibited activities for all the four, three, and two hydrolytic enzymes, respectively. In line with the current study, Mohammad et al. (16) reported that 10%, 40%, and 10% of the isolates isolated from Saudi Arabian hot springs produced five, three, and two of the extracellular enzymes, respectively. Likewise, Benammar et al. (41) also confirmed that 65.87% of bacterial isolates showed at least five enzymatic activities, while 18.43% of the isolates exhibited four enzymatic activities. In a related study, Ranawat and Rawat (43) also reported that 20% of the isolates isolated from Tapovan kund and Soldhar hot Springs in India produced all the three tested enzymes. The ability to produce two or more enzymes by microorganisms helps them survive in hostile environments as it helps them acquire nutrition by the breakdown of a variety of nutrients (43).

In the current study, of the total of 252 bacterial isolates, 45 of the isolates were selected as potent bacterial isolates for enzyme production based on the highest clear zone ratio. A statistically significant difference in the clear zone ratio for amylases was observed in two isolates: JUW103 and JUS2 with a clear zone ratio of 4.92 ± 1.13 and 4.58 ± 0.73 mm, respectively. Comparable to the current findings, Latorre and co-workers (21) reported that three of the screened isolates showed a significantly higher clear zone ratio (6.3, 6.1, and 5.8 mm) for amylase in comparison to other bacterial isolates. Another study by Yassin and colleagues (44) also indicated that three isolates produced the largest ratio of halo diameter (5.96 ± 0.057, 4.96 ± 0.057, and 4.73 ± 0.115 mm) for amylase than for the other isolates.

Morphological and biochemical test results in this study revealed that all the 45 bacterial isolates were rod-shaped, Gram-positive, catalase-positive, endospore formers, and motile and identified to the genus *Bacillus*. Similar to this result, Baltaci et al. (45), Panosyan et al. (34), and Yassin et al. (44) reported that all bacteria isolated from hot springs were Gram-positive, rod-shaped and motile, and endospore formers, possibly belonging to the genus *Bacillus*. In line with our finding, *Bacillus* is the most widely isolated genus in different hot springs studied worldwide (34, 45, 46). The reason for members of the genus *Bacillus* to become a common inhabitant of diverse extreme habitats is its spore-forming capacity and also production of a number of biocidal metabolites/enzymes (47). The abundance of *Bacillus spp*. could also be explained by its ability to migrate at extremely high rates, and the spores are known to resist different environmental stresses (48).

In this study, among the selected 45 isolates, some isolates were found capable of growing at extreme physiological conditions. It was observed that 50°C to 55°C was the optimal growth temperature, 7–8 was the optimal growth pH, and 3–10% was the optimal concentration of NaCl for cell growth. In line with this observation, Benammar and co-workers (41) reported that around 50% of the bacteria from the hot springs in Algeria could grow at 30°C, with the optimal growth temperature of 55°C, and all bacterial isolates were halotolerants. In addition, the optimal pH for the growth of the strains was neutral to slightly alkaline, ranging between 6.5 and 8.5. The related result was also reported by Panosyan et al. (34), in which the optimal pH for the growth of all isolates was 7, but the optimum temperature for their growth was 60°C, which is higher than that in the current study. In contrast to this, Ulucay et al. (49) reported that the optimum growth temperatures for most of the isolates were between 55°C and 65°C, and most isolates have show optimum growth ranges pH 5.0–9.5 and NaCl concentration range from 0 to 9%.

The 45 bacterial isolates from the four hot springs in this study were identified into three genera (*Bacillus, Paenibacillus,* and *Brevibacillus*) and five species (*B. licheniformis, B. cereus, P. thiaminolyticus, P. dendritiformis*, and *B. borstelensis*). The most dominant genus from the hot spring sites was *Bacillus,* and *B. licheniformis* was the most dominant species. Similarly, Sahay and co-workers (15) reported that from all identified bacteria that belong to 14 different genera, *Bacillus*, *Brevibacillus,* and *Paenibacillus* were the three dominant genera, and also *B. licheniformis*, *B. cereus*, *P. thiaminolyticus*, and *P. dendritiformis* were among the most identified species from the hot springs of Manikaran and Yumthang hot springs in the Indian Himalayas. Kumar and Sharma (50) also identified *P. dendritiformis, B. borstelensis,* and *Bacillus subtilis* from the hot springs located in the Yamunotri landscape of Garhwal Himalaya. In line with the current study, Ulucay and colleagues (49) also reported that *B. licheniformis* and *Bacillus subtilis* were the most dominant species obtained from the researched hot spring sources. Furthermore, another similar study of hot springs of Armenia investigated that the genus *Bacillus* was the most abundant bacteria identified from all hot springs assessed in the study, and the predominant *Bacillus* species was *B. licheniformis* (34). This is because *B. licheniformis* is known to be a spore-forming bacterium that is extensively distributed as a saprophytic organism in the environment (51).

### Conclusions

The finding of the current study revealed that Woliso, Shalla*,* and Wondo Genet hot springs are rich in potential thermophilic bacteria capable of producing extracellular hydrolytic enzymes, such as amylases, proteases, cellulases, and lipases. According to the enzyme assays, a majority of the isolates have the capability of producing more than one enzyme. Morphological and biochemical characterization revealed that all of the isolates in this study belong to *Bacillus* spp. With MALDI-TOF-MS and 16S rRNA sequencing, the bacterial isolates were identified as *Bacillus*, *Paenibacillus, and Brevibacillus* genera. The three genera were classified into five species: *B. licheniformis*, *B. cereus*, *P. thiaminolyticus*, *P. dendritiformis*, *and B. borstelensis*. Thermophilic bacteria identified in this study are best candidates for the production of thermostable enzymes that can be used for different industrial applications.
